# Perspectives on Deep Brain Stimulation and Its Earlier Use for Parkinson’s Disease: A Qualitative Study of US Patients

**DOI:** 10.3390/brainsci10010034

**Published:** 2020-01-08

**Authors:** Laura Y. Cabrera, Karen Kelly-Blake, Christos Sidiropoulos

**Affiliations:** 1 Center of Ethics and Humanities in the Life Sciences, Michigan State University, East Lansing, MI 48824, USA; kellyka1@msu.edu; 2 Department of Translational Neuroscience, Michigan State University, Grand Rapids, MI 49503, USA; 3 Department of Medicine, Michigan State University, East Lansing, MI 48824, USA; 4 Department of Neurology & Ophthalmology, Michigan State University, East Lansing, MI 48824, USA; sidirop3@msu.edu

**Keywords:** deep brain stimulation, patients’ perspectives, Parkinson’s disease, earlier use

## Abstract

Background: Deep brain stimulation (DBS) is being used earlier than was previously the case in the disease progression in people with Parkinson’s disease (PD). To explore preferences about the timing of DBS, we asked PD patients with DBS whether they would have preferred the implantation procedure to have occurred earlier after diagnosis. Methods: Twenty Michigan-based patients were interviewed about both their experiences with DBS as well as their attitudes regarding the possible earlier use of DBS. We used a structured interview, with both closed and open-ended questions. Interviews were transcribed verbatim and analyzed using a mixed-methods approach. Results: We found that the majority of our participants (72%) had high overall satisfaction with DBS in addressing motor symptoms (mean of 7.5/10) and quality of life (mean of 8.25/10). Participants were mixed about whether they would have undergone DBS earlier than they did, with five participants being unsure and the remaining nearly equally divided between yes and no. Conclusion: Patient attitudes on the early use of DBS were mixed. Our results suggest that while patients were grateful for improvements experienced with DBS, they would not necessarily have endorsed its implementation earlier in their disease progression. Larger studies are needed to further examine our findings.

## 1. Introduction

Most Parkinson’s disease (PD) patients initially respond well to treatment with L-dopa and dopamine agonists but, after five to seven years, the therapeutic effect of medication declines [1,2,3]. As a result, the most common subsequent medical intervention in the US has been deep brain stimulation (DBS), a neurostimulation intervention known to relieve PD motor symptoms and improve quality of life.

In the past, patients had to wait for several years after diagnosis to become a DBS candidate, as such the intervention was considered only for advanced PD and, in some cases, as a last resort treatment. However, over time, DBS has come to be regarded as a suitable treatment option earlier in the disease progression, once patients are no longer benefitting from medication [4], or for those who suffer from significant medication side effects [5]. Researchers are currently investigating the potential benefit of DBS as a disease-modifying strategy, prior to the onset of motor complications [6,7,8]. The timing of DBS initiation continues to raise a key question: How early is “too” early? Since patients make the final decision about receiving the intervention, they are key stakeholders regarding the timing of DBS. Thus, assessing patients’ attitudes about DBS and its initiation can inform treatment practices.

### Patient Attitudes Regarding DBS

There have been a number of studies looking at PD patient attitudes and experiences with DBS as well as factors influencing the decision to undergo DBS [9,10,11,12,13,14,15]. In an interview study, Haahr and colleagues [9] found that DBS patients experienced a phase of reconciliation, with many expressing difficulties during the adjustment of stimulation and medication. Another interview study by Hariz and colleagues found that patients highly appreciated the positive impact of DBS on their daily life, even if such an impact was time limited or brought side effects [12]. Maier et al. [13] noted that even when participants experienced significant motor function improvement, not all reported a subjective positive outcome.

Regarding factors influencing the decision to undergo DBS, Haahr et al. [10] reported that in advanced PD patients, the experience of living with and managing unpredictability made the decision to accept DBS relatively easy, with several participants viewing DBS as a ‘last resort’. Another important factor in the decision to accept DBS was having heard of reports of patients’ experiences with the treatment. Hamberg et al. [15] reported that patients’ knowledge and positive attitudes about DBS significantly influenced their decision making.

Few studies have reported on attitudes on earlier use of DBS. A Swedish study by Sperens et al. [14] explored knowledge and reasoning about DBS in patients who may have had reasons to consider DBS as a treatment alternative. They found that many patients were knowledgeable about DBS, expressing well-considered, balanced attitudes towards its outcome, yet they did not endorse an earlier implementation of DBS, considering DBS as an option only when oral medication was no longer sufficient.

Another study [7] focused on DBS perspectives of US patients potentially eligible for clinical research in early stage PD. The majority (72%) surveyed indicated that they would consider learning more about participating; among their reasons, they cited possibility of disease modification or slowing disease progression. For those not interested in such a study, reasons cited were avoidance of surgery and a perceived unfavorable risk–benefit ratio.

The current literature on patient attitudes and experiences regarding DBS for PD is far from comprehensive, with a majority of studies coming from European countries with health care systems and cultural backgrounds significantly different from the United States. Such differences likely influences patient attitudes and experiences. Moreover, the literature addressing the question of earlier DBS use generally is quite limited. The aim of this pilot study was to explore patients’ attitudes regarding the timing of DBS to help inform a future national survey with a larger sample of PD patients.

## 2. Methods

### 2.1. Design and Patient Recruitment

We conducted a pilot study in Michigan to explore the treatment experience of PD patients with DBS and their attitudes about earlier use of DBS.

Using a convenience sample, we interviewed twenty PD patients with DBS between October 2017 and April 2018. Patients were recruited via flyers left both at the clinics of previously identified movement disorder neurologists working in a high DBS volume practice in Michigan as well as in the practice of one of the authors (CS). Additionally, we posted online advertisements on both the Parkinson’s Association of West Michigan and on the Michigan State University Center for Ethics websites. All interested participants who had been diagnosed with PD and had undergone DBS were contacted via phone to explain the purpose of the interview and to schedule the interview. A consent form was e-mailed to all identified eligible participants prior to the interview.

We used a structured interview format with closed and open-ended questions. Interview questions were based on prior research [16] and on discussions with other researchers. All interviews were conducted by LC via Zoom online conferencing service or in person. Each interview lasted 30–45 min, was digitally recorded and transcribed verbatim. The interview included questions such as “How satisfied are you with DBS in terms of successfully addressing your PD motor symptoms?”, “How satisfied are you with DBS in terms of successfully addressing your quality of life?” Patients gave a number from 0–10 (0 = not at all satisfied to 10 = very satisfied) and were then asked to expand on the reasons for their response. We also asked them how they heard about DBS and recorded the number of years lapsed between their diagnosis and the clinician’s DBS recommendation. Regarding earlier DBS, we asked them “If given the choice, would you have agreed to have DBS implanted earlier in the progression of your disease?” and asked them to elaborate on their response.

### 2.2. Analysis

We employed a mixed-methods approach. A mixed-methods approach employs both qualitative and quantitative methods for the collection, assessment, and analysis of data. This approach allowed us to compare participant responses to the closed questions and the open-ended ones, the later provided context and background for the closed question responses. Interviews were analyzed using a qualitative content analytic approach [17,18,19]. Descriptive statistics were used to report demographics and to compare responses to closed questions. All subjects signed informed consent documentation prior to the interview. This study received IRB approval from Michigan State University (IRB# x17-1144e).

## 3. Results

Patient age at interview ranged from 53 to 71 years old. The duration of PD before surgery varied between 3 and 17 years and, at time of interview, patients had received DBS between 1 and 17 years (Table 1).

### 3.1. Satisfaction and Quality of Life

The majority of our participants were overall highly satisfied with DBS addressing both motor symptoms (mean of 7.6/10) and quality of life (mean of 8.4/10) (Table 2).

Patients who were very satisfied with DBS addressing motor symptoms mentioned that DBS had helped with their symptoms and facilitated enhanced mobility. One patient dissatisfied with DBS mentioned that he was not sure whether it was because DBS was not working or whether it was simply the result of disease progression. Those patients for whom DBS had failed to address their symptoms, suggested that it was a struggle accepting that nothing more could be done as programming changes had been exhausted.

In terms of quality of life, one very satisfied patient mentioned “I was about ready to give up in life” (P20). Patients who were not very satisfied mentioned that DBS was helpful at the beginning but that it stopped benefiting them.

### 3.2. Knowledge Sources

Patients accessed a variety of source material for information and knowledge acquisition including the Internet (60%, *n* = 12) (the Michael J Fox Foundation webpage, and associated patient testimonials were specifically mentioned as part of this), their doctors (50%, *n* = 10), and prior communication with another DBS patient(s) (45%, *n* = 9). Less common sources of information and knowledge included a newsletter published by a Parkinson’s patient society, information shared by a friend or family member, through their occupation or magazines. Many mentioned the benefits of having had previous contact with patients who had DBS as a way of clarifying expectations, but several respondents stated that this opportunity was not offered or facilitated by their clinicians. One participant asked the doctor about DBS after having read and watched programs about it.

### 3.3. Timing of DBS

**Figure 1:**
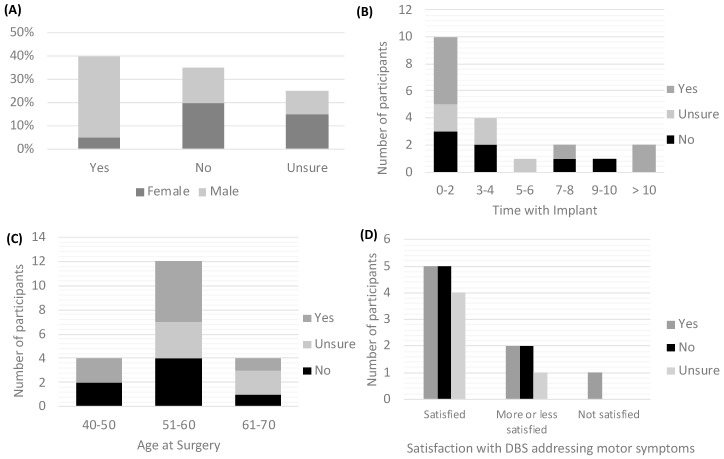
Factors driving patients’ responses about having DBS earlier. (**A**) Percentage of responses by gender; (**B**) Number of participants’ responses by time with implant; (**C**) Number of participants’ responses by age at DBS surgery; (**D**) Number of participants’ responses by level of satisfaction with DBS addressing motor symptoms.

In spite of the overall satisfaction with DBS, participants were mixed about whether they would have undergone DBS earlier than they did with 25% being unsure and the remaining divided between yes (40%) and no (35%) (Table 3). Level of satisfaction with motor symptoms improvement did not matter for patients reporting whether or not they would have undergone DBS earlier. Participants responding that they were ‘satisfied’ and those responding that they were ‘more or less satisfied’ answered ‘yes’ and ‘no’ to the question about having DBS earlier. Among those who said yes, most were male (87.5%), have had their implant for no more than two years (62.5%) and were between 51 and 60 years old at the time of the surgery (62.5%) (Figure 1).

For patients who answered ‘no’, the majority still agreed that the timing was important. For example, one patient explained, “You think about the bumps in your head and you think about the stimulator in your chest and it’s not like nothing, you know, it’s something that makes you pause and think about, but still I think it’s a good idea—I don’t regret having it. I, I would hate to get desperate for it” (Patient 01). Table 3 lists the participants’ reasons mentioned.

Among the responses of those patients who were unsure about earlier timing of DBS they explained that they were not emotionally ready, that they decided to pursue DBS only with declining medication effectiveness, and that they were unsure of earlier timing because of the involved risks. Another mentioned the ability to pay as an important consideration in making such a decision.

### 3.4. Neuroethical Concerns

The most common concern mentioned was fear of surgery. Associated fears included (1) fear about the surgery itself with the concern that someone was going in to manipulate inside their brain, (2) fear about the possibility of something going wrong and being left more debilitated, and (3) fear about being awake during the surgery. Some participants were concerned about having their personality changed by DBS. This concern, however, was mitigated after speaking to their clinical team, hearing others’ experiences, and weighing the risks and benefits. When asked about concerns related to personality change and DBS, one patient responded that she was not concerned with changes to her personality as a result of DBS because “It’s the same thing with pills” (Patient 01). Regarding DBS impact on patients’ social life and relationships, almost half (47%, *n* = 8/17) answered that they did not think earlier DBS would have any impact in those areas. One-third (34.3%, *n* = 6/17) answered that earlier DBS would have a positive impact on their social life and relationships, and the remainder were unsure.

## 4. Discussion

The aim of this pilot study was to explore experiences and attitudes about DBS timing among patients with PD. A majority of our participants expressed very positive outcomes with DBS in spite of side effects, in terms of motor improvements and quality of life. Participants were divided regarding whether they might have considered receiving DBS earlier. Many regarded DBS as a serious surgical procedure done on the brain and considered it a last resort only when medications were no longer effective. The main concern was fear of surgery.

### 4.1. Patients’ Experiences and Attitudes with DBS

In contrast with results reported by Haarh et al. [9], our participants did not experience trouble adapting to a changed body nor with difficulties in stimulation adjustment. Only a few indicated that their expectations of DBS had not been met. Similarly, our participants did not report as previous studies have found [9,20] that they had been liberated from the illness as a result of DBS nor did they consider the stimulation to be a “magic” process that would entirely free them from their physical impairment.

Most of our patients were aware that they still had the disease and they reported that DBS had helped them only with controlling motor symptoms. This attitude towards DBS was perhaps related to having been well informed by their DBS team on this particular issue. Yet, participants mentioned the idea of more freedom with DBS, as they were able to drive again and do activities such as exercising that they had enjoyed before getting PD.

Previous research has suggested that the experience of DBS for patients and spouses alike is influenced by their hopes and expectations of what surgery will enable them to achieve or regain in terms of goals [21]. The high level of satisfaction we found, even when some of our participants had developed side effects or did not have their expectations fully met, reflects findings in previous studies [12,22]. While it is well-recognized that axial motor symptoms might not respond and could even worsen after DBS [23,24,25], for our participants, the reduction of pre-operative motor symptoms of PD appears to outweigh the potential negative side effects on those areas. However, there have been reports where participants, in spite of positive clinical improvements, express dissatisfaction [26,27]. For example, Gilbert et al. reported that patients who felt alienated by their illness before the surgery continued to experience increased self-estrangement after the surgery [28]. Thus, more research in this area is needed with particular attention to understanding factors driving patient satisfaction and experiences with DBS among US PD patient populations.

In terms of negative experiences with DBS, our participants expressed some frustration with adjustments in stimulation parameters in order to establish an ‘optimal’ setting. Mathers et al. report similar findings [21].

### 4.2. Points of Entry and Information Regarding DBS

We found that the Internet and neurologists played a key role in referring patients and served as main sources of information for patients. Christen et al. report comparable findings in which private practice neurologists were the decisive “entry point” to DBS; additionally, up to 53.1% of patients used the Internet as their source of information [26]. The public uses the Internet to engage in social media interactions. In a recent study, Gardner et al. describe how YouTube has become an important social media platform in which patients create, share and consume health-related content. They illustrate how the representations of DBS in YouTube may have contributed to unrealistic expectations among the public and potential DBS recipients [29]. When offered by their neurologists most of our respondents agreed to DBS, with a minority hesitating and opting to wait. While other studies [15] describe patients’ individual initiative as crucial for having surgery, we found that to be the case in only a minority of our respondents.

Additional information entry points, such as offering to pair potential DBS candidates with existing DBS recipients, occurred in only a few cases. Many patients who were not afforded this opportunity mentioned that this would have been beneficial. It is important that clinicians not only discuss expectations and realistic benefits with the patient before surgery, but it could also help to have a panel of patients available who have already undergone the procedure to discuss their own experiences. This opportunity could help DBS candidates develop a more realistic picture of the range of possibilities they might expect with DBS. Engaging such a panel could enhance DBS candidates’ understanding of the “technical” information provided by clinicians (see reference [20]).

### 4.3. Patients’ Views on Earlier DBS

Our results reflect previous contrasting findings from Sperens et al. [14], where most patients would postpone DBS as long as possible compared to those by Heusinkveld [7], suggesting that many patients would consider participating in a trial for testing early DBS. In our study, we found that those participants with a positive view on earlier DBS felt that DBS should be the next step, when medication is no longer effective in controlling the symptoms of the disease. Others expressed hope for earlier DBS that might halt the disease progression, so that they might have more years ahead with a better quality of life. Such positive attitudes towards earlier DBS mirror those found in the study of Heusinkveld et al. [7]. A perceived unfavorable risk–benefit ratio and being satisfied with medication control of PD symptoms mitigated against early DBS, as found in our study and also as reported by Heusinkveld et al.

The fact that almost an equal number of patients raised negative views regarding earlier use of DBS, or were unsure, is at odds with the recent findings in the literature that favor proposing DBS for patients even earlier [8,30,31,32] in the disease progression. Given the small sample of patients, it would be unwise for us to generalize at this point, but these factors necessitate future research.

### 4.4. Patients Ethical Concerns with DBS

Mirroring findings in previous studies [14], one of the most frequent concerns expressed by our participants was the worry related to the neurosurgical procedure and its related risks.

Another important concern in the neuroethical literature is that of personality changes. Christen et al. [26] found that approximately 10% of their sample reported fear of personality changes. We found a similar percentage of patients reporting that fear, specifically prior to surgery. Commonly that concern dissipated once patients had a more in-depth discussion with the clinician. Given that the brain is considered to be the organ that orchestrates bodily functions, behaviors and thoughts, it is not surprising that people consider invasive interventions such as DBS as having a potential impact on their personality. Moreover, historical lessons from previous brain interventions such as lobotomies, wherein testimonials and the media have reported personality changes, might still loom large in the public’s mind and serve to shape patients’ concerns related to personality changes.

While other studies have mentioned as a concern the impact of DBS on patient’s social relationships, our participants did not perceive DBS as having an impact on such relationships, and one patient expressed that it had been beneficial.

### 4.5. Study Limitations

This study has several limitations. First, our findings are not generalizable, since all participants in this study were non-Hispanic White/Caucasian residing in Mid-Michigan and are thus not representative of all possible DBS patients. Second, most of our participants were reasonably knowledgeable about the disease and had an overall high level of education. Third, at the time of the interviews, many were very active in the PD community, helping others garner information about both the disease and treatment modalities. Fourth, each participant answered the semi-structured interview questions based on their own experiences and attitudes, which provides context and richness to the data but makes analyzing comparisons between respondents difficult. Lastly, and most importantly, we were unable to assess other possible confounders such as PD severity/stages, PD clinical subtype, motor symptoms at the time of DBS, indication for DBS, DBS targets, and symptom response to DBS because this was an exploratory qualitative semi-structured interview study and we did not conduct a patient chart abstraction. Notwithstanding these limitations, this study is an important contribution to the examination of patients’ reasons for or against the earlier use of DBS. More research is needed to examine patients’ perceptions and attitudes about earlier use of DBS, and various factors shaping those perceptions.

## 5. Conclusions

This qualitative study shows that, if earlier DBS initiation had been an available option, DBS patients are divided about whether they would have made a different decision. Our results suggest that while patients are grateful for improvements experienced with DBS, they did not endorse its implementation early in the disease progression. This is true even after adjusting for time with implant, age at surgery and satisfaction with DBS addressing motor symptoms. We also found that male patients are more likely to agree to having DBS earlier in the disease progression compared to female patients. The key reason for agreeing to earlier DBS was trust in their doctor’s recommendation that DBS would be beneficial by possibly slowing disease progression. Reasons patients gave for not agreeing to earlier DBS were (1) they did not consider themselves ill enough, and (2) they felt that medications were still working for them.

The main concerns expressed by our participants diverge from those most emphasized in the neuroethics DBS literature. More research with a larger sample is needed to better understand what factors might shape patients’ willingness to support earlier DBS, and to examine their associated values and preferences regarding this treatment.

## Figures and Tables

**Table 1:** Socio-demographic and clinical characteristics in 20 patients with Parkinson’s disease.

Gender Men/Women	*N* (%) 12(60)/8(40)
**Age** Age at diagnosis Duration of disease (years) before surgery Age at deep brain stimulation (DBS) surgery Age at interview Time between surgery and interview (years)	**Median (range)** 49 (35–63) 6.5 (3–17) 56.5 (46–69) 60 (53–71) 2.5 (1–17)
**Civil status** Married	***N*(%)** 18(90)
**Race** Non-Hispanic white	***N*(%)** 20(100)
**Level of education** High school Continuing education Bachelor Master	***N*(%)** 2(10) 1(5) 8(40) 9(45)
**Employment status at time of DBS** Working full time Working part time Off work/ retired	***N*(%)** 15(75) 1(5) 4(20)
**Member of a Parkinson’s disease (PD) society** Yes/No	***N*(%)** 12(60)/8(40)
**Overall Impact of DBS** (0 mark deterioration–10 mark improvement)	**Mean ± SD (range)** 8.6 ± 1.72 (5–10)

**Table 2:** Level of satisfaction, years after diagnosis and views on getting DBS earlier than they did.

**Satisfaction with DBS addressing motor symptoms** Satisfied More or less satisfied No satisfied	***N*(%)** 14(70%) 5(25%) 1(5%)
**Satisfaction with DBS addressing quality of life** Satisfied More or less satisfied No satisfied	***N*(%)** 17 (85%) 3 (15%) 0
**Years after diagnosis that doctor suggested DBS** NA, patient brought it up More than 7 years after diagnosis 5–6 years after diagnosis 3–4 years after diagnosis At diagnosis-2 years after	***N*(%)** 2 (10%) 6 (30%) 4(20%) 6(30%) 2(10%)
**Would have agreed to had DBS earlier than he/she had it** Yes No Unsure	***N*(%)** 8 (40%) 7 (35%) 5 (25%)

**Table 3:** Reasons given by patients regarding having agreed/not agreed to have DBS before.

Yes	No
“you get to feeling that you’re headed downhill and it’s kind of like a low-hanging fruit.” (Patient 02) “the shakes would have gone lot sooner. If they have said “oh yeah okay we can do this for you right away’ I would’ve done it, but I didn’t have that choice.” (Patient 03) “I probably would’ve listened to my doctor. So I can’t say I would’ve gone against them and done it, anyways” (Patient 09) “If the doctor felt it would help, I would have do it” (Patient 13) “To continue doing my exercise” (Patient 16) “It’s possible. … seven months before I had (DBS) done, that things really were getting out of hand” “there’s a possibility that if you had done earlier it could slow the disease down” (Patient 18) “I” was having side effects, because of them trying so many different Parkinson’s drugs you know…we would have chosen to have (DBS) without having to try any of those drugs” (Patient 19) (Patient 20)	“Earlier I was well controlled with medication” (Patient 01) “…knowing what I know now, I would probably have waited. I would’ve look for more individuals who have had the procedure” (Patient 05) “I would say probably not because there are many other options. I felt, um, when I chose DBS that I had exhausted most of the other approaches to address my particular symptoms” (Patient 10) “I just wasn’t in that frame of mind. I was not ready” (Patient 12) “I don’t think I really realized the impact of the disease until they mentioned it to me” (Patient 14) “No, I think I did it about the right time.” (Patient 15) “I wasn’t as bad off as I had looked” (Patient 17)
